# Lineage-specific loss of the type VI secretion system in *Acinetobacter baumannii* ST19 is associated with reduced accessory genome content

**DOI:** 10.1128/aac.01938-25

**Published:** 2026-07-01

**Authors:** Adam Valcek, Diana Isabela Costescu Strachinaru, Kristina Nesporova, Patrick Soentjens, Anke Stoefs, Charles Van der Henst

**Affiliations:** 1 Microbial Resistance and Drug Discovery, VIB-VUB Center for Structural Biology, VIB, Flanders Institute for Biotechnology 82116, Brussels, Belgium; 2 Structural Biology Brussels, Vrije Universiteit Brussel (VUB) 70493, Brussels, Belgium; 3 Center for Infectious Diseases, Queen Astrid Military Hospital 82012, Brussels, Belgium; 4 Department of Biology, Microbial Systems Cell Biology Group, KU Leuven 26657, Leuven, Belgium; 5 Department of Clinical Sciences, Institute of Tropical Medicine 567788 , Antwerp, Belgium; 6 Department of Microbiology, Queen Astrid Military Hospital 82012, Brussels, Belgium; 7 Department of Microbiology, Universitair Ziekenhuis Brussels, Brussels, Belgium; Universita degli Studi di Roma La Sapienza, Rome, Italy

**Keywords:** antimicrobial resistance, *Acinetobacter baumannii*, Type VI secretion system, accessory genome, comparative genomics

## Abstract

The type VI secretion system (T6SS) is a widespread bacterial nanomachine involved in interbacterial competition and environmental adaptation. Its distribution and evolutionary significance in *Acinetobacter baumannii* is not fully understood. Here, we investigated the ST19 lineage, revealing a near-complete absence of the T6SS across 159 genomes from diverse geographic and clinical origins. Comparative genomic analysis showed that the T6SS locus in ST19 is frequently disrupted, with some isolates harboring insertions of mobile genetic elements, while in others, the region is fragmented or unresolved. To assess whether T6SS loss is associated with broader genomic changes, we analyzed 8,218 publicly available *A. baumannii* genomes. Interestingly, T6SS-negative genomes did not exhibit increased acquisition of accessory genetic material. Instead, they carried fewer antimicrobial resistance genes (8.06 vs 10.56 per genome), fewer virulence genes (101.8 vs 132.5 per genome), and comparable plasmid replicon content relative to T6SS-positive genomes. However, T6SS-negative genomes exhibited a higher number of insertion sequences (IS) per genome compared to T6SS-positive genomes (mean ~12.3 vs ~9.0 IS elements per genome, respectively). Ancestral state reconstruction of the *frmRAB* and the putative chlorite dismutase genes within ST19 revealed identical distribution patterns and strong phylogenetic conservation, with 7 inferred state transitions indicating limited evidence for independent gain or loss events. Our results suggest that T6SS loss represents a lineage-specific evolutionary trajectory characterized by loss of the T6SS locus and reduced accessory genome, suggesting an alternative ecological strategy.

## INTRODUCTION

*Acinetobacter baumannii* is an emerging opportunistic nosocomial pathogen that causes a variety of infections, including urinary tract infections, wound infections, meningitis, ventilator-associated pneumonia, bloodstream infection, and endocarditis (1). *A. baumannii* is a member of *Enterococcus faecium*, *Staphylococcus aureus*, *Klebsiella pneumoniae*, *Acinetobacter baumannii*, *Pseudomonas aeruginosa*, and *Enterobacter* spp. (ESKAPE) pathogens (2), and due to its frequent resistance to last-line antibiotics such as carbapenems, it has been designated as a critical priority pathogen by the World Health Organization for research and development of new antibiotics (3, 4).

Besides conventional antibiotics, *A. baumannii* can withstand challenging environmental conditions such as oxidative stress, prolonged periods of desiccation, human serum, and disinfectants (5).

The ability of *A. baumannii* to acquire exogenous DNA via natural competence (6) and conjugation (7) contributes to the high plasticity of its genome (6). Horizontal gene transfer plays a significant role in the evolution of *A. baumannii* lineages, resulting in a number of highly successful clonal lineages such as sequence type 1 (ST1), ST2, ST10, ST15, ST25, ST79, and ST85 (Pasteur scheme) (8, 9). While ST1 and ST2 are responsible for the majority of infections caused by extensively drug-resistant *A. baumannii*, some clones are specific to certain geographical locations (9), such as ST25 (Europe, United Arab Emirates, South America, and Asia) (10), ST32 (South Korea, Europe, the United States, Canada, and the Middle East) (11), or ST78 (Italy, Ukraine, and the United States) (12).

While horizontal gene transfer, especially plasmid transfer via conjugation, can shape entire lineages and their success (13), an active type VI secretion system (T6SS) poses a unique challenge to conjugative plasmids, as *Acinetobacter* plasmid donors and recipients may kill each other (14). The T6SS machinery is usually encoded in a single chromosomal locus (15). The T6SS of *A. baumannii* has been shown to mediate the killing of *Escherichia coli*, *K. pneumoniae*, and *P. aeruginosa*, while different strains of *A. baumannii* are also able to kill each other (15, 16).

In this study, we performed a comprehensive genomic analysis of ST19 isolates alongside a large comparative data set of 8218 *A. baumannii* genomes. We have assessed genetic determinants disrupting the T6SS-encoding locus in a lineage of *A. baumannii* ST19, the occurrence of which is increasing regionally (Ukraine and Georgia) (17). Furthermore, we have performed comparative genomic analysis of the global population of *A. baumannii* ST19, as well as on T6SS-negative non-ST19 lineages. We have evaluated the relationship between T6SS presence and accessory genome content, including antimicrobial resistance genes, virulence-associated genes, plasmid replicons, and insertion sequences. Finally, we used phylogeny-informed ancestral state reconstruction to investigate the evolutionary distribution of selected lineage-associated traits.

Our results demonstrate that T6SS loss in ST19 is not associated with increased acquisition of accessory genetic material. Instead, T6SS-negative genomes exhibit a relatively reduced accessory genome, suggesting that the evolutionary trajectory of this lineage reflects a distinct ecological strategy.

## RESULTS

### *A. baumannii* ST19 strains and genomes

A concerning report on the increasing occurrence of *Acinetobacter baumannii* ST19 encoding carbapenemases was recently published (17), raising questions about the genomic characteristics of this lineage. Three isolates of *A. baumannii* ST19 were obtained from two different patients (patient 1: wound, rectal swab; patient 2: rectal swab) and long-read whole-genome sequenced and assembled *de novo*.

**Fig 1:**
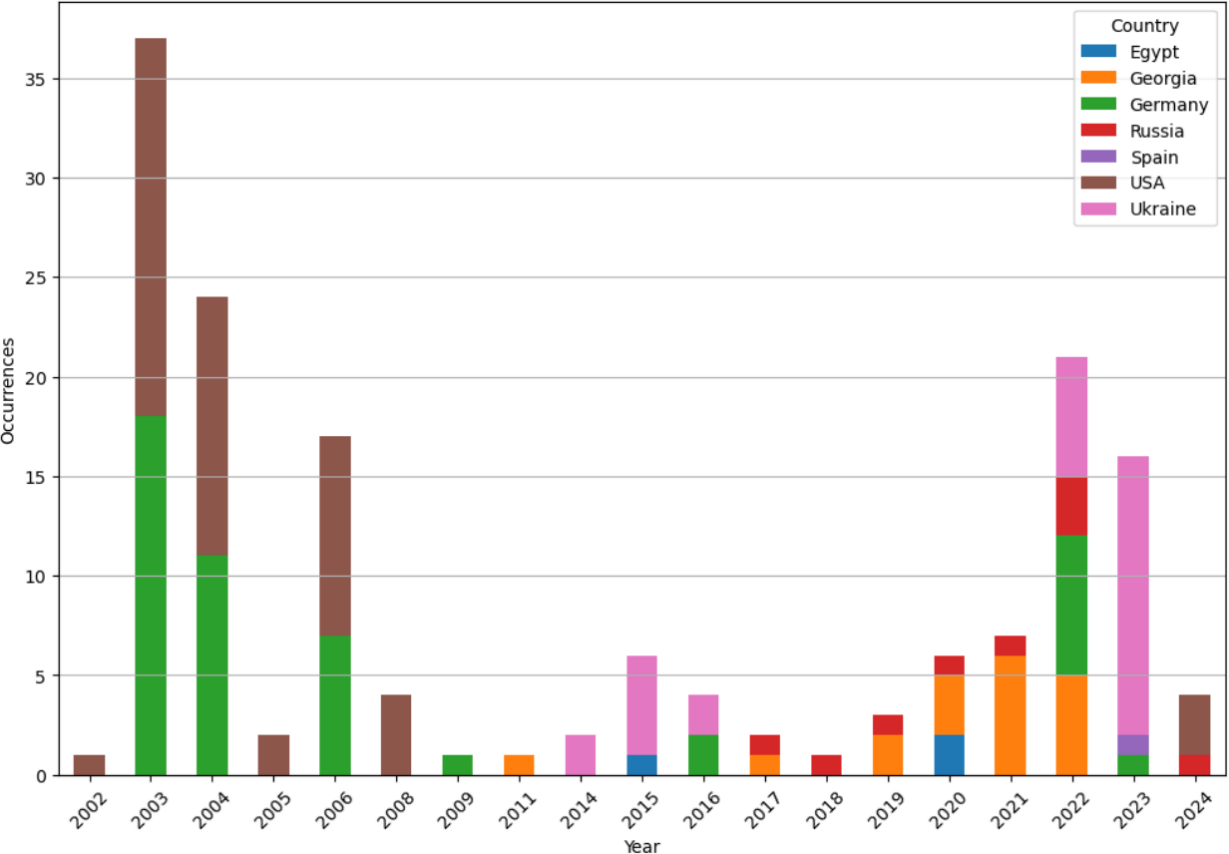
Temporal and spatial occurrence of *A. baumannii* ST19 based on genomes deposited in public repositories and available metadata.

A further 156 genomes (including 97 from Luo et al. [17]) of *A. baumannii* ST19 were retrieved from public repositories (GenBank and BIGSdb). The occurrence of *A. baumannii* ST19 in publicly available repositories coincides with periods of military conflicts (Fig. 1), such as the Iraq War (2003) and the war in Ukraine (2022). However, we acknowledge that this might also be caused by sampling bias.

The examined genomes carried a variety of virulence and resistance genes (Fig. S1), including resistance to last-resort antibiotics such as carbapenems. One-third (*n* = 53) of the 159 genomes described in this study encoded at least one carbapenemase gene (*bla*_OXA-23_, *bla*_OXA-72_, *bla*_NDM-1_, or *bla*_NDM-5_), with 6 encoding 2 and 9 encoding 3 carbapenemases. Furthermore, a broad variety of plasmid replicons was detected (Fig. S1).

Genomic analysis identified the presence of multiple partial transposons, including ΔTn*9* (18) carrying chloramphenicol resistance gene and ΔTn*10* (19) carrying the tetracycline resistance gene. Interestingly, 61/159 (38.4 %) genomes of *A. baumannii* ST19 carried putative chlorite dismutase and formaldehyde resistance (*frmRAB*) genes, with 98.4% amino acid identity to *Massilia glaciei* and 96.7% to *Ideonella dechloratans*, where it combats oxidative stress (20). To our knowledge, this is the first time putative chlorite dismutase has been reported in the genome of *A. baumannii*.

Furthermore, these transposons disrupted and replaced the locus encoding the T6SS. The entire region of T6SS deletion exhibits extensive genetic rearrangements, as ΔTn*9* and ΔTn*10* were acquired and disrupted by IS*26* and IS*4321*, and by IS*26*, respectively.

### Genetic variability of the *A. baumannii* ST19 lineage

**Fig 2:**
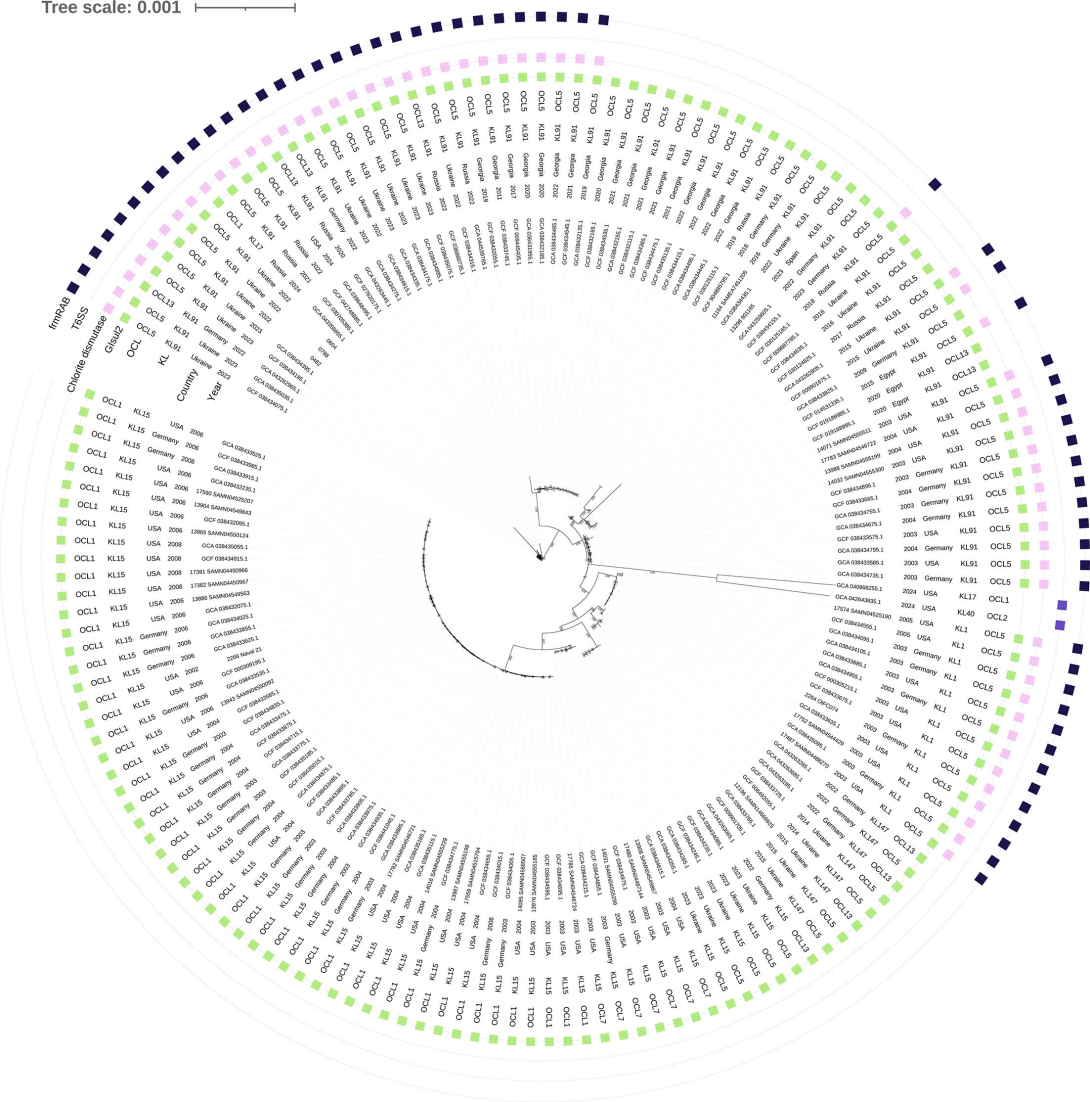
Phylogenetic analysis of 3 isolates and a further 156 of *A. baumannii* ST19 genomes, depicting year of isolation, country, capsule locus type (KL), lipooligosaccharide outer core (OCL) type, and presence of GI*sul2* genetic island, putative chlorite dismutase, T6SS, *frmRAB* genes, respectively.

The whole-genome sequences were subjected to genotyping (sequence type, capsule locus [KL] type, lipooligosaccharide outer core locus [OCL] type, antimicrobial resistance, and virulence genes). A phylogenetic analysis was performed on these isolates, including genomes of the same ST from public repositories (GenBank and BIGSdb) (Fig. 2; Fig. S1).

While various Multilocus Sequence Types (Pasteur scheme) such as ST2 or ST1 often belong to various KL types (6), the ST19 lineage exhibited limited variability with 69/159 genomes being KL91, 66/159 KL15, followed by KL1 (13/159), KL147 (8/159), KL17 (2/159), and KL40 (1/159). Interestingly, 53/55 OCL1 encoded KL15 (2/55 OCL1 were KL17); the most predominant OCL type, OCL5 (*n* = 87), was variable and encompassed KL91, KL1, KL147, and KL15. Furthermore, all of the genomes of KL15/OCL1 formed one large, genetically related cluster (Fig. 2).

The vast majority (157/159) of *A. baumannii* ST19 harbored GI*sul2*, a chromosomally carried Genomic Island encoding sulfonamide resistance determinant (*sul2*) along with putative arsenic resistance genes (21). We have detected a range of 0 to 6 plasmid replicons (22) in the genomes of *A. baumannii* ST19, with the rP-T1 replicon being the most prevalent (*n* = 122/159), followed by r3-T23 (*n* = 46/159), r3-T12, and r1-T8 (*n* = 44/159 both). Interestingly, 18 isolates did not carry any detectable plasmid replicon. However, the *Acinetobacter* typing schemes are still being developed (22), possibly, undetected (or below the detection threshold) plasmids must be considered as well.

**Fig 3:**
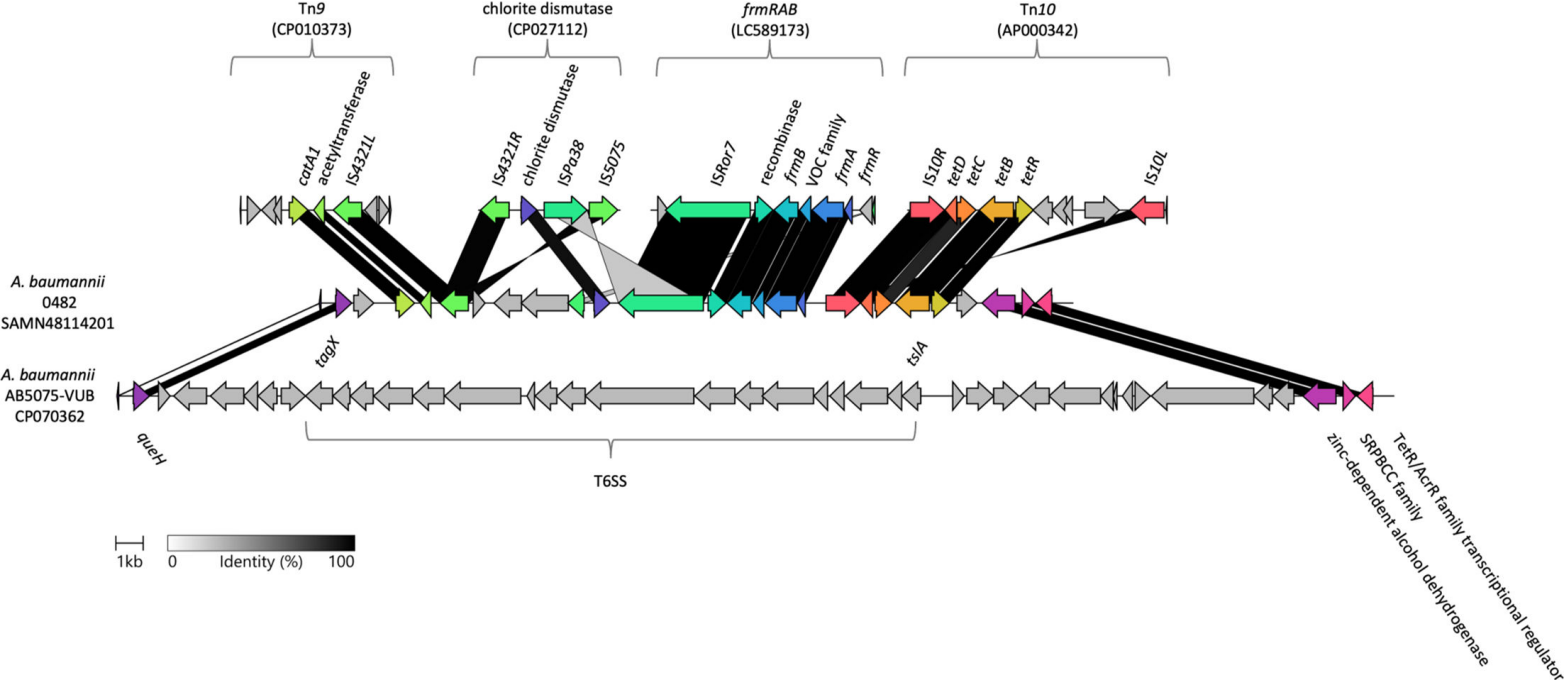
Depiction of the T6SS locus disruption of *A. baumannii* ST19 (0482-SAMN48114201) as a representative genome, compared to AB5075-VUB (CP070362) showing deletion mediated by ΔTn*9*, putative chlorite dismutase, putative formaldehyde resistance locus *frmRAB*, and ΔTn*10*. The alignments are based on amino acid identity.

By evaluating the T6SS locus in the ST19 lineage of *A. baumannii*, we identified variability in the genetic context of the deletion of these genes. Of 61/159 genomes carrying putative chlorite dismutase and formaldehyde resistance genes in the locus of T6SS, 55/61 harbored ΔTn*10* carrying the tetracycline and ΔTn*9* carrying chloramphenicol resistance genes (Fig. 3). Five putative formaldehyde resistance and chlorite dismutase-encoding genomes (5/61) did not harbor ΔTn*10* carrying the tetracycline resistance, and 1/61 did not harbor ΔTn*9* carrying chloramphenicol resistance genes. However, mapping on the reference genome (0604-SAMN46706488) confirmed that in all these genomes (61/159), these genetic rearrangements were associated with disruption of the T6SS locus.

Interestingly, 2/159 genomes of *A. baumannii* ST19 (GCA_042643835.1 and GCA_040868255.1) still encoded a likely functional T6SS. However, compared to AB5075-VUB (accession no. CP070362), a number of single nucleotide polymorphisms (SNPs) (99% pairwise identity over 19,417 bp) was detected in these two genomes, while the broader genetic neighborhood only sporadically exhibited SNPs. The genomes GCA_042643835.1 and GCA_040868255.1 belonged to KL40/OCL2 and KL17/OCL1, respectively, which are rare for this lineage (a sole KL40 and one of two KL17, respectively). While none of the SNPs were non-sense, the influence of missense or synonymous (e.g., via transfer RNA) on the functionality of T6SS remains to be explored.

### The loss of the T6SS in non-ST19 lineages of *A. baumannii*

The presence of the T6SS locus was assessed in 8,218 genomes of *A. baumannii* available in BIGSdb. The lack of the T6SS was detected in 1,287 genomes (including 159 genomes of *A. baumannii* ST19). The most dominant lineage of *A. baumannii* lacking the T6SS in this comparison was ST2 (*n* = 435; 33.7%), followed by ST19 (*n* = 159; 12.3%), and ST94 (*n* = 82; 6.4%) (Fig. S3A and B).

The phylogenetic comparison of all 1,287 genomes (Fig. S2) showed clustering of all *A. baumannii* ST19 and 103 non-ST19 genomes together. The non-ST19 genomes in the ST19 cluster consisted of 93 single-locus variant (SLV) sequence types, including one novel ST, and 10 double-locus variant sequence types, including one novel ST (Fig. S3C).

The presence of *frmRAB* and putative chlorite dismutase-encoding genes was detected predominantly in *A. baumannii* ST19 but also in two *A. baumannii* ST315 genomes within the same cluster. A single genome (ST2525) outside of the ST19 cluster was found to carry the *frmRAB* genes.

### T6SS absence is not associated with increased accessory genome content

To determine whether loss of the T6SS is associated with increased acquisition of accessory genetic material, we compared antimicrobial resistance genes, virulence-associated genes, plasmid replicons, and insertion sequences across 8,218 *A. baumannii* genomes.

All analyzed genomes carried at least one antimicrobial resistance gene. However, T6SS-negative genomes carried fewer antimicrobial resistance genes compared to T6SS-positive genomes (*n* ≈ 7,088) (mean 8.06 vs 10.56).

Similarly, T6SS-negative genomes carried fewer virulence genes (mean 101.8 vs 132.5).

Plasmid replicon content was comparable between the T6SS-negative and T6SS-positive genomes (2.74 vs 3.03 replicons per genome). However, the T6SS-negative genomes exhibited a higher number of IS per genome compared to T6SS-positive genomes (mean ~12.3 vs ~9.0).

This suggests that T6SS absence is not associated with increased acquisition of resistance determinants, virulence genes, or plasmid content; however, T6SS-negative genomes display higher plasticity.

### Phylogenetic conservation of *frmRAB* and putative chlorite dismutase within *Acinetobacter baumannii* ST19 lineage

Ancestral state reconstruction was performed to assess the evolutionary distribution of the *frmRAB* and the putative chlorite dismutase genes within the ST19 lineage. Both markers showed identical distribution patterns across the phylogeny, with co-occurrence observed in all isolates where they were detected. An equal-rates model was supported over an all-rates-different model by Akaike Information Criterion corrected for sample size (ΔAICc = 2.05). Ancestral state reconstructions indicated strong support for a single dominant state across the phylogeny, with most internal nodes and terminal isolates assigned high posterior probabilities and 7 inferred state transitions on the maximum a posteriori reconstruction. This pattern is consistent with predominantly vertical inheritance, with limited evidence for additional independent gain or loss events of *frmRAB* or putative chlorite dismutase gene.

These results support the hypothesis that these loci are sub-lineage-associated features within ST19 and share an evolutionary background.

## DISCUSSION

*Acinetobacter* has the core components of the T6SS conserved and found in a single chromosomal locus (15). The *A. baumannii* strains use Type VI Secretion System (T6SS) to kill non-kin *A. baumannii* and other bacterial species such as *E. coli*, *K. pneumoniae*, and *P. aeruginosa* (16, 23). The active T6SS can be a challenge to conjugation and horizontal gene transmission (15).

The Tn*10* was originally identified and described in *Shigella flexneri* (19). This corresponds to the best match of the ΔTn*10* nucleotide sequence in this study, which was detected in *Salmonella enterica* serovar Typhi and *S. flexneri*. The ΔTn*10* observed in this study has the highest similarity to regions from *Enterobacter hormaechei* IncHI2 plasmids (>99% nucleotide identity and 97% query coverage). Furthermore, the ΔTn*9* (24) examined in this study was BLASTn-detected (with >99% nucleotide identity and 100% query coverage) in *Klebsiella pneumoniae* IncF plasmids.

Although *adhC* genes facilitating resistance to formaldehyde, which are native to *Acinetobacter*, have been reported (25), we have identified a locus, likely originating from an *Enterobacter hormaechei* IncHI2 plasmid, or a *Klebsiella pneumoniae* (or *quasipneumoniae*) IncF plasmid (95% nucleotide identity and 100% coverage). However, the origin of the putative chlorite dismutase gene remains undeciphered, while the BLASTn of the putative chlorite dismutase gene observed in this study results in identical hits (>96% nucleotide identity and 98% query coverage) in various species, such as *P. aeruginosa* (chromosome), *S. enterica* (IncHI2 plasmid), *K. pneumoniae* (IncF plasmid), and *E. hormaechei* (chromosome), as well as *Vibrio rumoiensis* (plasmid), *Raoultella ornithinolytica* (IncF plasmid), and *Leclercia adecarboxylata* (IncHI2 plasmid).

A total of 61/159 genomes were identified to encode the same genetic cargo (ΔTn*9,* ΔTn*10*, putative chlorite dismutase, and the putative formaldehyde resistance cluster) as the one present in 3 strains of ours (0788, 0482, and 0604) in the region where the T6SS locus was expected. For the rest of the genomes (98/157; as 2 genomes carried the T6SS locus) not encoding T6SS machinery, we could not identify the underlying mechanisms of loss due to draft-genome assemblies and possible crucial information missing. However, the absence of genetic cargo expected to replace the T6SS locus could suggest either a different way of T6SS locus displacement or further genomic rearrangement, possibly leading to the formation of further sub-clades of the *A. baumannii* ST19 lineage. Alternatively, the sub-clade with no detectable T6SS locus perhaps never encoded the T6SS in the first place, or may represent alternative evolutionary scenarios that cannot be resolved with the available data.

Of the total 1,287 genomes, 179 different sequence types of *A. baumannii* lacking the T6SS were detected. Furthermore, 35 genomes were assigned novel STs containing at least 1 novel allele. As the typing scheme is still under development and allelic profiles were not manually curated in this study, some of these novel STs may represent redundant assignments. While additional non-ST19 *A. baumannii* lineages were detected that lack the T6SS, the specific displacement and acquisition of the ΔTn*9*, ΔTn*10*, *frmRAB*, and putative chlorite dismutase gene combination was observed only in *A. baumannii* ST19. The lineages *A. baumannii* ST19 (except for two genomes) and ST94 (SLV of ST19) consistently lacked detectable T6SS. While the most genomes lacking the T6SS were detected in the *A. baumannii* ST2 lineage, this is skewed by the overrepresentation of this lineage in the data set from BIGSdb (*n* = 6,402), and the T6SS might have been replaced or lost, as observed in other cases of clinical isolates lacking some or all T6SS genes (26, 27).

The *A. baumannii* lineage ST94 (SLV of ST19) completely lacked the T6SS; however, no *frmRAB* or putative chlorite dismutase genes were detected in this lineage. Unfortunately, the lack of complete chromosomal sequences of *A. baumannii* ST94 lineage from this data set limits the ability to resolve the genomic context of T6SS loss.

The loss of the T6SS in entire lineages of *A. baumannii* may represent a lineage-associated genomic feature with potential epidemiological relevance. While there were reports of sporadic loss of part or the entire T6SS locus in *A. baumannii* clinical isolates (28, 29), the lineage-wide loss could be an indicator for the detection of a difficult-to-treat lineage, widely resistant to disinfectants and antibiotics, with high persistence potential.

While the T6SS activity can be repressed by a plasmid (14), recent work has shown that T6SS activity in *Acinetobacter baumannii* can also be regulated at the post-transcriptional level via small RNA-mediated repression, rather than solely through genomic loss (30). In this context, attenuation of T6SS function by regulatory suppression or irreversible gene loss supports a model of an evolutionary trade-off, in which reduced interbacterial antagonism may be offset by increased ecological flexibility or compatibility with horizontal gene transfer.

### Conclusion

The *Acinetobacter baumannii* ST19 lineage shows a lineage-specific absence of the Type VI Secretion System (T6SS) and specific accessory genome composition. Comparative genomic analyses of the large data set showed reduced accessory gene content (plasmid replicons, antimicrobial resistance, or virulence-associated genes) but increased genome plasticity (insertion sequences).

The absence of T6SS within ST19 suggests a shared evolutionary background, and while the plausible consequence is a fitness trade-off in a specific environment, the mechanisms and consequences of this loss remain to be fully understood.

## MATERIALS AND METHODS

### Bacterial isolates, genomes, and metadata

Three isolates of *A. baumannii* ST19 were obtained from two different patients (patient 1: wound, rectal swab; patient 2: rectal swab). A further 156 genomes (including 97 from Luo et al. [17]) of *A. baumannii* ST19 were retrieved from public repositories (GenBank and BIGSdb) as of November 2025. In order to analyze other lineages of *A. baumannii* for T6SS locus deletion, all available genomes of *A. baumannii* in BIGSdb (*n* = 8,218) were downloaded and screened.

### DNA extraction and whole-genome sequencing

The DNA was extracted using DNeasy Blood & Tissue Kit (QIAGEN). A rapid barcoding kit (V14) (ONT) was then used for library preparation, which was then subjected to long-read whole-genome sequencing using the R10.4.1 flowcell with the PromethION (P2 Solo) platform by Nanopore.

### Genome assembly and annotation

The long-read sequences were basecalled using the SUP model and adaptor- and quality-trimmed (*Q*  ≤  15) using Porechop v0.2.2 (https://github.com/rrwick/Porechop) and NanoFilt v2.8.0 (31), respectively. The reads were then assembled using Flye 2.9.3 (32).

### Phylogenetic analysis

The maximum-likelihood trees were constructed using gff files generated by Prokka (33) for the core-genome alignment created by Roary (34). RAxML (35) was used for the calculation of the phylogenetic tree under the GTRGAMMA model of rate heterogeneity, supported by 500 bootstraps. The tree was visualized in iTOL (36).

### Identification of resistance determinants

The resistance and virulence genes were detected using ABRicate (https://github.com/tseemann/abricate), employing ResFinder 4.0 (37) and (38) databases, respectively, with a 90% threshold for both gene identity and coverage. The typing of capsule-encoding loci (KL) and lipooligosaccharide outer core loci (OCL) was determined using Kaptive (39). The ABRicate (https://github.com/tseemann/abricate) was used to detect the presence of plasmid replicons using the AcinetobacterPlasmidTyping database (https://github.com/MehradHamidian/AcinetobacterPlasmidTyping) (21), while the genes encoding GIs*ul2*, putative chlorite dismutase, putative formaldehyde resistance genes, and T6SS-encoding genes were detected using a custom database.

### Comparative analysis of accessory genome content in T6SS-positive and T6SS-negative genomes

To assess the relationship between T6SS presence and accessory genome content, a data set of 8,218 publicly available *Acinetobacter baumannii* genomes was analyzed. Genomes were divided as T6SS-positive or T6SS-negative. Antimicrobial resistance genes were identified using ABRicate with the ResFinder database. Virulence-associated genes were detected using ABRicate with the VFDB database. Plasmid replicons were identified using MOB-suite (v3.1.9). Insertion sequences were identified using ABRicate with the ISfinder database.

For each genome, the total number of database hits was used as a proxy for accessory gene content. Results are reported as gene hits per genome. Mean values were calculated for T6SS-positive and T6SS-negative groups and compared.

### Ancestral state reconstruction

The evolution of the *frmRAB* and the putative chlorite dismutase genes within the *Acinetobacter baumannii* ST19 lineage was assessed using ancestral state reconstruction on a phylogenetic tree of ST19 isolates. Trait presence was encoded as binary characters (0 = absence, 1 = presence) based on genotype (isolates lacking these genes were treated as absent for this analysis). Both *frmRAB* and the putative chlorite dismutase gene showed identical presence/absence distribution across all genomes and were therefore combined into a single binary trait vector. The phylogeny and trait data sets only included overlapping isolates. Ancestral state reconstruction was performed in R using the corHMM package v2.8. Equal-rates and all-rates-different continuous-time Markov models were fitted and compared using Akaike Information Criterion corrected for sample size (AICc). Marginal posterior probabilities of ancestral states were used to evaluate the evolutionary distribution of both traits across the phylogeny, and the number of state transitions inferred from the maximum a posteriori reconstruction was determined.

### Genome quality assessment

Genome assemblies were assessed for completeness and contamination using CheckM (v2). Only genomes meeting quality thresholds (≥95% completeness and ≤5% contamination) were retained for downstream analyses. This threshold allowed us to use all but one genome (GCA_040868255.1), where contamination represented 10.63%; however, as it represented one of two T6SS-positive genomes of the *A. baumannii* ST19 lineage, we decided to retain it.

## Data Availability

The whole-genome sequencing data were deposited in GenBank under the following accession numbers: strain 0788-SAMN46706486, strain 0482-SAMN48114201, and strain 0604-SAMN46706488.

## SUPPLEMENTAL MATERIAL

Fig. S1 aac.01938-25-s0001.eps Phylogenetic tree of all *A. baumannii* genomes available in BIGSdb that do not encode the T6SS.

Fig. S2 aac.01938-25-s0002.eps Phylogenetic analysis of three isolates and an additional 156 *A. baumannii* ST19 genomes.

Fig. S3 aac.01938-25-s0003.tiff (A) Quantification of the 20 most prevalent STs of *A. baumannii* genomes from BIGSdb lacking the T6SS. (B) Pie chart showing the percentage distribution of the 10 most prevalent STs of *A. baumannii* genomes from BIGSdb lacking the T6SS. (C) Numeric prevalence of STs within the cluster containing the ST19 lineage shown in Fig. S1.

Supplemental material aac.01938-25-s0004.docx Supplementary Figures' legends

ASM does not own the copyrights to Supplemental Material that may be linked to, or accessed through, an article. The authors have granted ASM a non-exclusive, world-wide license to publish the Supplemental Material files. Please contact the corresponding author directly for reuse.
