# Supplementary material for: Lineage-specific loss of the type VI secretion system in Acinetobacter baumannii ST19 is associated with reduced accessory genome content
Source: Antimicrob Agents Chemother. 2026 Jul 1;70(8):e01938-25. doi: 10.1128/aac.01938-25 (PMC13436141; doi:10.1128/aac.01938-25)
Supplement: Supplementary file 1 [file aac.01938-25-s0004.docx]

Supplementary Figure 1: Phylogenetic tree of all *A. baumannii* genomes available in BIGSdb that do not encode the T6SS. The cluster containing the ST19 lineage is highlighted; turquoise indicates ST19, and gray indicates non-ST19.

Supplementary Figure 2: Phylogenetic analysis of three isolates and an additional 156 *A. baumannii* ST19 genomes, depicting the year of isolation, country, capsule locus type (KL), outer core lipooligosaccharide type (OCL), and the presence of the GI*sul2* genomic island, putative chlorite dismutase, T6SS, the *frmRAB* operon, plasmid replicons, and resistance and virulence genes, respectively.

Supplementary Figure 3: A, Quantification of the 20 most prevalent sequence types (STs) of *A. baumannii* genomes from BIGSdb lacking the T6SS. B, Pie chart showing the percentage distribution of the 10 most prevalent STs of *A. baumannii* genomes from BIGSdb lacking the T6SS. C, Numeric prevalence of STs within the cluster containing the ST19 lineage shown in Supplementary Figure 1.
